# Nonoperative treatment of Achilles tendon rupture with 6- versus 8-week protocol: a single-center cohort study from Finland

**DOI:** 10.2340/17453674.2026.46872

**Published:** 2026-09-30

**Authors:** Ville HAPPONEN, Anu KOIVISTO, Anssi HÄRKÖNEN, Heikki KRÖGER

**Affiliations:** 1 Department of Orthopaedics, Traumatology and Hand Surgery, Kuopio University Hospital, Kuopio; 2 University of Eastern Finland, UEF, Kuopio; 3 Kuopio Musculoskeletal Research Unit, University of Eastern Finland, Kuopio University Hospital, Kuopio, Finland

## Abstract

**Background and purpose:**

Although nonoperative treatment of Achilles tendon rupture (ATR) has become increasingly common, the optimal duration of immobilization and early functional rehabilitation remains uncertain. We aimed to compare outcomes of 8-week and 6-week nonoperative treatment protocols for ATR, with particular focus on re-rupture rates and treatment failure requiring surgery.

**Methods:**

This register study included all university hospital-treated ATRs in the Northern Savo region, Finland, between 2014 and 2023. Patients were identified using ICD-10 and NOMESCO procedure codes. We compared outcomes between an 8-week nonoperative treatment protocol used in 2014–2018 and a 6-week protocol used in 2019–2023. Outcomes included patient demographics, treatment distribution, indications for surgery, and complications.

**Results:**

Among 479 ATRs, 429/479 (90%) were treated nonoperatively. Operative treatment was mainly done in athletes. Operative treatment was performed in 32/251 (13%) patients in the 8-week protocol group and in 18/228 (7.9%) patients in the 6-week protocol group. Re-rupture rates were similar between the 8-week and 6-week protocols (7/251 [2.8%] vs 4/228 [1.8%] absolute risk difference −1.0 percentage point, 95% confidence interval −3.7 to 1.6). Operative treatment was mainly indicated for athletes, chronic ruptures, and re-ruptures, with a postoperative complication rate of 14%.

**Conclusion:**

Outcomes regarding re-rupture and treatment failure following the 6-week nonoperative protocol were comparable to those observed with the 8-week protocol However, larger comparative studies in more diverse populations are needed to confirm the generalizability of these findings.

Achilles tendon rupture (ATR) is a common injury with a globally increasing incidence [1]. According to Leino et al., the incidence of ATR in Finland increased from 17.3 to 32.3 per 100,000 person-years between 1997 and 2019 [1]. ATR predominantly affects male patients, with previously reported male-to-female ratios ranging from 3.2:1 to 4.0:1 [1–6]. Sports activities are the primary injury mechanism for ATR [1]. there has been a clear decline in surgical treatment over the past 2 decades [1,6,7]. Although surgical treatment is associated with a lower risk of re-rupture than nonoperative treatment, it carries an increased risk of nerve injury and deep or superficial infections [8,9]. Furthermore, recent studies have reported similar patient-reported outcomes following surgical and nonoperative management of ATR [9].

Despite the widespread use of nonoperative management for ATR, the optimal duration of immobilization and functional rehabilitation remains debated.

The primary aim of our study was to compare the outcomes of 2 nonoperative treatment protocols for ATR of 8 and 6 weeks. We evaluated associated re-rupture rates and treatment failure requiring surgery. Secondary objectives were to describe nonoperative and operative treatment patients’ demographics, associated comorbidities, shifts in treatment strategies, the indications for surgical treatment, and complications associated with operative treatment.

## Methods

### Study design and data source

This register study was performed in the population of Northern Savo (approximately 250,000 people) in Finland. Most ATRs are treated at Kuopio University Hospital (KUH) because primary health care providers refer these patients to the hospital emergency department or orthopedic outpatient clinic for diagnosis and treatment. Patients with ATR treated in private hospitals were not included in this study. Therefore, some younger patients, for example professional athletes seeking private care, may not have been captured by the study.

We identified all hospital-treated patients with ATR between 1 January 2014 and 31 December 2023 using KUH electronic medical records. There was no direct or indirect contact with patients (including electronic or telephone communication).

This study was reported in accordance with the Strengthening the Reporting of Observational Studies in Epidemiology (STROBE) guidelines.

### Population

We used the ICD-10 code S86.0, which is specific for Achilles tendon injury, to identify all ATR cases. Operatively treated ATRs were identified using a combination of diagnosis and procedure codes. We used the ICD-10 code S86.0 together with the Nordic Medico-Statistical Committee (NOMESCO) procedure codes NHL10 (suture or reinsertion of the Achilles tendon), NHL30 (suture or reinsertion of a tendon of the ankle or foot), NHL68 (tendon lengthening, shortening, or fixation in the ankle or foot), and NHL99 (other operations on muscles or tendons of the ankle or foot). Indications for surgery and complications were extracted directly from the patients’ medical records.

### Outcomes

We stratified the register-based numbers of ATRs managed either nonoperatively or operatively by year, sex, and recorded comorbidities that may predispose individuals to ATR and may also influence treatment selection. The following comorbidities were included: diabetes mellitus with or without chronic complications, peripheral vascular disease, hypertension, and inflammatory polyarthritis that had been treated in hospital inpatient or outpatient settings within 2 years before the ATR. Postoperative complications were categorized into 4 classes: infections, thromboembolic complications, nerve-related complications, and re-ruptures. All recorded wound-healing problems were associated with postoperative infections and were therefore included in the infection category. Failed nonoperative treatment was defined as cases in which primary conservative management did not result in satisfactory tendon healing or functional recovery and subsequently required surgical treatment

### Statistics

All statistical analyses were performed using IBM SPSS Statistics, version 29 (IBM Corp, Armonk, NY, USA). Categorical variables are presented as numbers and percentages of patients or procedures, as appropriate. Continuous variables are expressed as mean and standard deviation (SD). Fisher’s exact test was used to compare categorical variables, and the Mann–Whitney U test was used for nonparametric continuous variables. Analyses focused on estimation of between-group differences and corresponding 95% confidence intervals (CI). For binary outcomes, results are presented as absolute risk differences with 95% CI.

### Ethics, registration, data sharing, use of AI, funding, and disclosures

The ethics review committee of the Hospital District of Northern Savo approved the study plan (6155/13.04/2024 [PSSHP]). Because of privacy restrictions, the data cannot be shared. The manuscript has been professionally language edited, although parts of the language revision were also assisted by AI-based language support tools. This research did not receive any specific grant from funding agencies in the public, commercial, or not-for-profit sectors. The authors report no conflict of interests. Complete disclosure of interest forms according to ICMJE are available on the article page, doi: 10.2340/17453674.2026.46872

## Results

479 patients with ATR were treated at Kuopio University Hospital between 2014 and 2023. Of these, 251 were managed under the 8-week nonoperative treatment protocol and 228 under the 6-week protocol (Figure 1, Table 1). Baseline characteristics of the 2 treatment groups are presented in Table 2. The mean age was 50.1 years (SD 15.3) in the 8-week protocol group and 50.9 years (SD 16.4) in the 6-week protocol group (Figure 2). Most patients in both groups were male and did not have the comorbidities under investigation. Among patients with acute ATRs, the most common indication for operative treatment (n = 50) was athlete status (28%). Other indications for surgery included ATR re-rupture (22%), chronic ATR (22%), and tendon transection or avulsion fracture of the calcaneus (10%).

**Figure 1:**
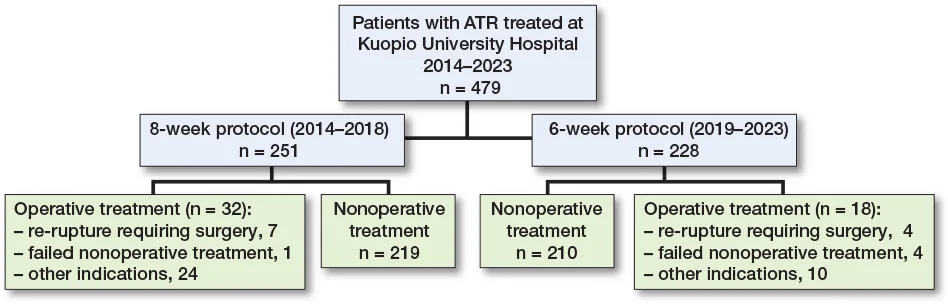
Study flow diagram.

**Table 1:** Nonoperative treatment protocol for Achilles tendon ruptures at Kuopio University Hospital

Protocol	Postoperative period	Treatment
**Until 2018 (8-week protocol)**		
Immobilization/orthosis		
Weeks 1–4: Equinus cast (extreme plantar flexion)		
Weeks 5–8: Functional orthosis allowing plantar flexion, preventing dorsiflexion beyond neutral position		
Weightbearing		
Weeks 5–8: Permitted as tolerated		
Rehabilitation		
At 8 weeks: Physiotherapy referral; heel lift recommended for 1–2 months		
**From early 2019 (6-week protocol)**		
Immobilization/orthosis		
Weeks 1–2: Equinus cast		
Weeks 3–4: Hinged orthosis locked at 15° plantar flexion		
Weeks 5–6: Hinged orthosis with free motion 0°–30° plantar flexion		
Weightbearing		
Week 1–6: Allowed as tolerated during orthosis use		
Rehabilitation		
At 6 weeks: Physiotherapy referral; heel lift recommended for 1–2 months		

**Table 2:** Baseline characteristics between 8‑week and 6‑week nonoperative management protocols for ATR. Values are count (%) or as specified

Factor	8-week protocol (n = 251)	6-week protocol (n = 228)	Standardized difference
Age, mean (SD)	50 (15)	51 (16)	–0.05
Male sex	198 (79)	184 (81)	–0.04
Diabetes mellitus	6 (2.4)	2 (0.9)	0.12
Hypertension	8 (3.2)	3 (1.3)	0.13
Peripheral vascular disease	1 (0.4)	0 (0.0)	0.09
Inflammatory polyarthritis	2 (0.8)	6 (2.6)	–0.15

**Figure 2:**
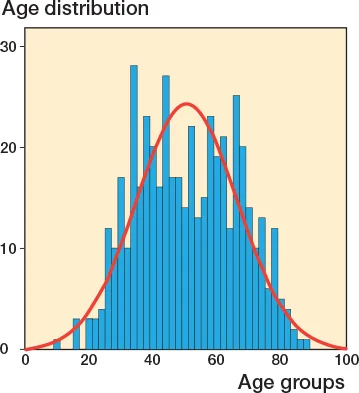
Age distribution of patients with Achilles tendon ruptures at Kuopio University Hospital, 2014–2023

### Outcomes

Operative treatment of ATRs was 13% (32/251) of patients treated with the 8-week protocol and 7.9% (18/228) of those treated with the 6-week protocol, corresponding to an absolute risk difference of −4.9 percentage points (CI −10.3 to 0.6) (Table 3). When comparing treatment protocols, the re-rupture rate requiring operative treatment was 2.8% (7/251) in the 8-week protocol group and 1.8% (4/228) in the 6-week protocol group, corresponding to an absolute risk difference of −1.0 percentage points (CI −3.7 to 1.6). Further operative treatment following failed primary nonoperative management was required in 0.4% (1/251) of patients treated with the 8-week protocol and 1.8% (4/228) of patients treated with the 6-week protocol, corresponding to an absolute risk difference of 1.4 percentage points (CI −0.5 to 3.2). In addition, 11 chronic ATRs were treated operatively during 2014–2023. Of these, 6 patients had missed ATRs and received no treatment initially. In these cases, physiotherapist-guided rehabilitation was attempted before surgical treatment.

**Table 3:** Comparative outcomes between 8‑week and 6‑week nonoperative management protocols for ATR. Values are count (%) or as specified

Factor	8-week protocol (n = 251) n (%)	6-week protocol (n = 228) n (%)	Between-group difference (CI), %
Operative treatment	32 (13)	18 (7.9)	–4.9 (–10.3 to 0.6)
Re-rupture requiring surgery	7 (2.8)	4 (1.8)	–1.0 (–3.7 to 1.6)
Failed nonoperative treatment	1 (0.4)	4 (1.8)	1.4 (–0.5 to 3.2)

CI = 95% confidence interval.

After operative treatment of ATR, the postoperative complication rate was 14%, and the mean age of these patients was 40 years (SD 11.9). Specifically, infections occurred in 8% of cases, re-ruptures in 2%, thromboembolic complications in 2%, and nerve complications in 2%.

## Discussion

We aimed to compare the outcomes of 8-week and 6-week nonoperative rehabilitation protocols for ATR, a comparison not previously reported. The principal finding was that shortening the duration of immobilization and early functional rehabilitation from 8 weeks to 6 weeks was associated with similar re-rupture rates of 2.8% vs 1.8%.

Regarding treatment failure requiring subsequent operative intervention, the observed frequency was 1.4 percentage points higher following the 6-week protocol and the confidence interval surrounding the between-group estimate was wide. This reflects considerable uncertainty due to the small number of events. However, the relatively small sample size limits the reliability of the statistical analysis.

In addition to the comparison of rehabilitation protocols, we observed a continued shift towards nonoperative treatment. Operative treatment rates decreased from 13% in the 8-week protocol period to 7.9% in the 6-week protocol period. This trend aligns with longitudinal data from Svedman et al. and Mattila et al., which document a steady decrease in surgery for ATR over recent decades [5,7].

Our population was dominated by males and largely free of the investigated comorbidities—hypertension, diabetes mellitus, peripheral vascular disease, or inflammatory polyarthritis—within 2 years before the injury. Only 5% of the patients in this study had comorbidities. This suggests that acute ATR remains primarily an injury of a relatively healthy and active population. Although systemic factors and comorbidities are recognized as predisposing risks, their low prevalence in our study highlights that mechanical loading and age-related tendon degeneration in otherwise healthy individuals remain the primary drivers of rupture.

In our study, the mean age of 51 years is slightly higher than reported in recent studies [6,10]. This may partly reflect the relatively old population structure of Northern Savo, and the inclusion of patients of all ages. The finding is also consistent with observations by Ho et al. regarding the increasing age of the ATR population over time [2].

Comparison of the 2 treatment protocols showed similar re-rupture rates. This finding is consistent with modern rehabilitation trends emphasizing shorter immobilization, earlier weightbearing, and early-stage exercises. Previous studies suggest that these approaches do not increase the risk of re-rupture while potentially facilitating rehabilitation and recovery [11,12]. Hutchison et al. [13] reported low re-rupture rates using a structured nonoperative rehabilitation program with prolonged immobilization and functional bracing, whereas Aujla et al. [14] reported favorable outcomes following a shortened rehabilitation protocol. In addition, a recent meta-analysis found no significant differences between early and delayed weightbearing regarding re-rupture rates or other adverse event [12].

When comparing re-rupture rates after nonoperative treatment with those reported in the literature, our finding of 3% is consistent with—or even lower than—rates reported in meta-analyses such as Ochen et al. [15] and randomized trials such as Myhrvold et al. [9].

The overall complication rate among operatively treated patients in this study was 14%, with infections occurring in 8% of cases. All patients undergoing operative treatment received preoperative antibiotic prophylaxis. The infection rate was relatively high in our study compared with the results reported by Ochen et al., whose meta-analysis indicated an incidence of 2.8% [15]. Re-ruptures, thromboembolic events, and nerve complications each occurred in 2% of operative cases.

### Limitations

First, we lack individual clinical data for all patients with ATR, including patient-related risk factors such as body mass index, smoking status, alcohol consumption, and comorbidities treated outside the hospital setting. Second is that some minor complications, such as superficial surgical site infections or deep venous thrombosis not requiring inpatient care, may not have been captured. Third is that we do not have patient-reported outcome measures. Fourth is the relatively high mean age of the study population. This may partly reflect the demographic structure of Northern Savo, but also the exclusion of patients treated in private healthcare, where younger and more active individuals may be overrepresented. Therefore, the study population may not fully represent all patients with ATR in the region. Fifth is the relatively small number of operative treatments, re-ruptures, and treatment failures.

### Conclusion

We showed that outcomes following the 6-week nonoperative rehabilitation protocol were comparable to those observed with the 8-week protocol regarding re-rupture rates and treatment failure requiring surgery. Re-rupture rates and treatment failure remained uncommon in both groups.

*In perspective,* our findings suggest that a shorter functional treatment strategy of 6 weeks may represent a safe and effective alternative to the traditional 8-week protocol. However, larger comparative studies in more diverse populations are needed to confirm the generalizability of these findings.

## Notes

VH: collection, analysis and interpretation of data, design of the study, drafting of the manuscript, and revision of the manuscript. AK: collection of data, drafting of the manuscript, and revision of the manuscript. AH: design of the study, and revision of the manuscript. HK: interpretation of data, design of the study, and revision of the manuscript.

Handling co-editors: Per Henrik Randsborg and Robin Christensen.

*Acta* thanks Ståle Bergman Myhrvold and Jacques Riad for help with peer review of this manuscript.
